# The bacterial microbiome and cancer: development, diagnosis, treatment, and future directions

**DOI:** 10.1007/s10238-024-01523-9

**Published:** 2024-11-28

**Authors:** Hasnaa H. Qasem, Wael M. El-Sayed

**Affiliations:** Department of Zoology, Faculty of Science, Ain Shams University, Abbassia, Cairo, 11566 Egypt

**Keywords:** Dysbiosis, Fecal transplantation, Genotoxin, Immune modulation, Inflammation, Oxidative stress, Prebiotics, Probiotics

## Abstract

The term "microbiome" refers to the collection of bacterial species that reside in the human body's tissues. Sometimes, it is used to refer to all microbial entities (bacteria, viruses, fungi, and others) which colonize the human body. It is now generally acknowledged that the microbiome plays a critical role in the host's physiological processes and general well-being. Changes in the structure and/or function of the microbiome (dysbiosis) are linked to the development of many diseases including cancer. The claim that because of their negatively charged membrane, cancer cells are more vulnerable to some bacteria than normal cells and that is how the link between these bacteria and cancer evolved has been refuted. Furthermore, the relationship between the microbiome and cancer is more evident in the emerging field of cancer immunotherapy. In this narrative review, we detailed the correlation between the presence/absence of specific bacterial species and the development, diagnosis, prognosis, and treatment of some types of cancer including colorectal, lung, breast, and prostate cancer. In addition, we discussed the mechanisms of microbiome–cancer interactions including genotoxin production, the role of free radicals, modification of signaling pathways in host cells, immune modulation, and modulation of drug metabolism by microbiome. Future directions and clinical application of microbiome in the early detection, prognosis, and treatment of cancer emphasizing on the role of fecal transplantation, probiotics, prebiotics, and microbiome biomarkers were also considered.

## Background

There are more than a thousand different bacterial species living in the tissues of the human body collectively known as the microbiome or microbiota. Although the two terms were used interchangeably in earlier literature, a distinction is now recognized between them. Microbiota refers to the community of microorganisms, including bacteria, viruses, fungi, and archaea, that inhabit a specific environment, such as the human gut. In contrast, microbiome encompasses not only the microbiota but also their collective genomes and the interactions between these microorganisms and their host. The human microbiome inhabits several anatomical regions including, but not limited to, the layers of skin, oral cavity, nasal passages, stomach, both large and small intestines, urinary system, and vagina [1]. However, many other studies define the term "microbiome" to encompass the assemblage of bacteria, viruses, fungi, and other microbial entities, which inhabit and colonize the human body [2]. Microorganisms are important in a multitude of biological processes and significantly impact human health and illness. The inception of the microbiome notion may be attributed to the esteemed Nobel winner, Joshua Lederberg, in 2001.

The discovery of the microbiome and its importance has opened new avenues of research in fields like medicine, nutrition, and environmental science. The significance of the microbiome in the host's physiological functioning and overall well-being is now generally acknowledged. The microbiome fulfills several metabolic functions and contributes to the proper defense system maturation [3]. The makeup of the normal microbiome exhibits inter-individual variation and is subject to the effect of several variables, such as anatomical site conditions, host genetic factors, dietary patterns, and the use of antibiotics [4]. Dysbiosis is described as a disturbance in the makeup and activity of the microbiota in individuals suffering from a certain disease, in comparison with healthy individuals. It could also be defined as an imbalance in the ratio of beneficial: harmful bacteria with significant increase of the latter [5].

Emerging evidence suggests that changes in the microbiome may play a role in the development of certain multifactorial chronic conditions, such as type 2 diabetes mellitus (T2DM) and chronic kidney disease (CKD). For example, alterations in the gut microbiota, including reductions in Lactobacillus species and short-chain fatty acid-producing bacteria, have been observed in individuals with T2DM, which may be linked to impaired insulin sensitivity and decreased dietary fiber intake [6]. Similarly, CKD has been associated with changes in the lower intestinal microbiota, notably reductions in Lactobacillaceae and Prevotellaceae groups [7]. Additionally, hemodialysis patients have been found to have lower levels of Bifidobacteria and higher levels of Clostridium perfringens [8]. While these associations provide valuable insights, further research is needed to clarify the precise mechanisms by which the microbiome may influence these conditions.

The microbiome governs health, and any change that occurs to it can lead to different diseases including cancer [9]. Empirical evidence strongly suggests that specific microorganisms initiate cancer by inducing genetic mutations through the action of genotoxins they produce, while others promote the progression of tumor cells by modulating the immune system [10]. Microbiomes can cause many types of cancer such as colorectal cancer, lung cancer, breast cancer, and prostate cancer as well as other diseases. Scientific research on the complex relationship between the human microbiota and cancer has become increasingly interesting.

## Microbiome and cancer

Cancer is characterized by uncontrolled cell proliferation and metastasis. Cancer research has primarily focused on genetic and environmental factors that cause cancer development. However, dysbiosis emerges as a factor with substantial impact on the cancer development. Dysbiosis affects metabolic, inflammatory, and immunological pathways [11], and could produce genotoxic metabolites [12]. The link between microbiome and development of colorectal cancer (CRC) is one of the first relations reported and hence the most extensively studied relation. The host immune system releases genotoxic agents in response to the metabolites synthesized by gut microbiome. The interaction between these metabolites and genotoxic agents causes CRC. The bacterial diversity and density isolated from intestinal mucosa and feces of CRC patients showed a significant reduction. Bacteria such as *Fusobacterium nucleatum* and *Escherichia coli* have been linked to the development of CRC [13].

Dysbiosis has been also linked to other types of cancer such as breast, lung, liver, and pancreatic cancer. These discoveries underscore the significance of the microbiome in the cancer biology domain. Moreover, the progression and success of cancer immunotherapy have emphasized this correlation between microbiota and cancer. The treatment outcomes have also been enhanced by the presence of specific bacterial species like *Akkermansia muciniphila* [14] and retarded by bacterial species like *Bacteroides fragilis* [15]. Our understanding of the link between microbiome, and cancer prognosis and treatment is a captivating area of research that will reshape the oncology as microbiome has been proved to be a substantial diagnostic and therapeutic target. Therefore, in this review we focused on the microbiome and different types of cancer such as colorectal, lung, breast, and prostate, in addition to the different mechanisms of microbiome–cancer interactions, as well as the future directions and clinical application of microbiome in the treatment of cancer.

### Microbiome and colorectal cancer (CRC)

In 2020, CRC accounted for 9.4% of all deaths reported in cancer victims, making it one of the most common cancer types that kill people. CRC has become more common in recent years [16]. The gut microbiomes are involved in important activities such as digestion, energy balance and metabolism, vitamin and nutrient synthesis, and immune function development and control. Additionally, it aids in the synthesis of many chemical compounds that subsequently enter the bloodstream, influencing diverse tissues and organs [17]. Furthermore, changes in the gut microbiome composition may arise because of many events, including dietary patterns, aging, geographical location, racial background, exposure to surrounding microorganisms, administration of antibiotics, occurrence of infectious diarrhea, or migration across international boundaries [16].

The microbiome in the development of colorectal cancer is considered more of a driver than a passenger. Emerging evidence suggests that specific microbial communities and their metabolites can actively contribute to carcinogenesis by influencing inflammation, altering local immune responses, and affecting the metabolism of dietary and endogenous compounds [18]. Although the relationship between the microbiome and colorectal cancer is complex and multifaceted, with interactions potentially varying between individuals, the current understanding supports the role of the microbiome in driving the progression of colorectal cancer rather than being a passive bystander. Recent findings showed that specific microbiota profiles and their metabolites contribute to the CRC development [19, 20]. Networks of pathobionts within the tumor mucosal environment are linked to specific tumor mutations and metabolic profiles. The research indicates that the presence of these pathobionts is associated with reduced survival rates, implying that they could affect disease advancement and patient outcomes. Consequently, this evidence favors the driver hypothesis over the passenger hypothesis [21].

The link between microbial abundance and cancer incidence demonstrates the importance of the gut microbiome in the CRC. Neoplastic transformation is mostly shown in the distal intestine, which harbors a high concentration of microbiota. The correlation between the presence or absence of bacteria and the development of cancer has been established in experimental animal models. Various scholarly papers investigating the involvement of colon bacteria in the CRC have consistently identified particular bacterial species such as *Fusobacteria*,* Porphyromonadaceae*,* Alistipes*,* Staphylococcaceae*,* Coriobacteridae*,* Akkermansia*, and *Methanobacteriales* as being constantly higher, despite variations in the methodologies used throughout these studies [22]. Yachida et al. observed metagenomic changes in the fecal microbiota in patients with colorectal neoplasms. They found that *Peptostreptococcus stomatis*, *Peptostreptococcus anaerobius*, *Parvimonas micra*, *Pneumocystidomycetes sp.*,* Saccharomycetes*, and *Fusobacterium nucleatum* were abundant in patients suffering from metastatic colorectal adenocarcinoma [23]. While other bacteria which have a protective role were consistently decreased, these include *Bifidobacterium*,* Clostridium*,* and Lactobacilli* [24].

Furthermore, while some microbial metabolites such as nitrogenous substances, poly- and mono-unsaturated fatty acids, and amino acids were continuously raised in the CRC patients [22, 25], some supplementary chemicals, such as butyrate, demonstrated a consistent decline as colonic cancer advanced. In a separate investigation, variations in the abundance of individual microbes were observed when comparing the mucosa of tumor and non-tumor regions in patients with CRC and those without. Notably, alterations in the microbiome and metabolome composition were evident at various stages of colorectal neoplasia, encompassing adenomatous polyps, early-stage cancer, and metastatic disease. This highlights the etiological and prognostic importance of the microbiota [9].

### Microbiome and lung cancer

Lung carcinoma has also been linked to changes in the diversity and intensity of the respiratory system microbiome [26]. In healthy people, the dominant bacteria in the respiratory system are *Proteobacteria*,* Firmicutes*,* Fusobacteria*,* Actinobacteria*, and *Bacteroidetes* [27], while *Pseudomonas*,* Modestobacter*,* Streptococcus*,* Prevotella*,* Veillonella*,* Haemophilus*, and *Neisseria* are the main types found in lung cancer patients’ respiratory tract [28]. *Selenomonas*,* Capnocytophaga*,* and Veillonella* were ubiquitous in patients with lung adenocarcinoma and squamous cell carcinoma [27].

Buccal microorganisms such as *Streptococcus* and *Wechsler* have been linked to the activation of the ERK and PI3K signaling pathways resulting to metastasis of lung cancer [27–29]. Infections by *Mycobacterium tuberculosis* and *Helicobacter pylori* help in the progression of lung cancer. The presence of *Bradyrhizobium japonicum* in the lower airway was only found in lung cancer patients [30].

There is also some evidence of a link between microbiome composition and a certain histologic type of tumor. *Acidovorax*,* Klebsiella*,* Rhodoferax*,* Comamonas*, and *Polarmonas* have been shown to be more prevalent in cases of small-cell carcinoma and are not often seen in instances of adenocarcinoma [1]. Admittedly, modifying bacterial composition in the respiratory tract can inhibit cancer development [28].

### Microbiome and breast cancer

Breast cancer is the second most often diagnosed cancer in women and the third most prevalent in men. Globally, breast cancer stands as the major cause of cancer-associated mortality [31]. A correlation between altered gut microbiota and breast cancer has been reported. A higher prevalence of *Clostridia*,* Enterobacterium*,* Lactobacilli*,* Bacteroides*, and *Escherichia coli* was reported in breast cancer patients [32]. Certain bacterial species, such as *Methylobacterium radiotolerans*, are more abundant in breast cancer cells compared to healthy cells. Patients with advanced stages of breast cancer have lower bacterial loads and reduced expression of antibacterial response genes underscore the role of dysbiosis in compromising the immune system's capacity to fight cancer cells. Therefore, assessment of bacterial load could serve as a biomarker for breast cancer diagnosis and staging [33].

The variety and composition of the gut microbiome impact the development of breast cancer through the regulation of circulating estrogen levels. Changes in the microbes responsible for the metabolism of estrogen may result in higher amounts of circulating estrogen and a higher risk of breast cancer [34, 35]. Enzymes such as β-glucuronidases produced by Bacteroidetes break down estrogen metabolites, allowing their reabsorption into the bloodstream through enterohepatic circulation [32]. The "estrobolome" refers to the group of bacterial genes in the intestine responsible for metabolizing estrogen and its by-products [36].

### Microbiomes and prostate cancer

Prostate cancer is the second type of cancer in incidence and the fifth highest contributor to cancer-related mortalities in men [37]. The progression of prostate cancer is affected by many factors such as exercise, nutrition, and obesity [37, 38]. The potential impact of the microbiome on the development and progression of prostate cancer may be attributed to a combination of direct and indirect interactions [39, 40]. Direct pathways are related to the prostate and urinary microbiomes, while indirect pathways are linked to the gastrointestinal microbiome [2, 41].

The correlation between the urine microbiome and the occurrence of chronic prostatitis, benign prostatic hyperplasia, and prostate cancer has been extensively studied [39]. Urine samples from 135 males were assessed using the 16S rRNA gene amplicon sequencing, prior to undergoing prostate biopsy. The data suggested that certain pro-inflammatory bacteria, such as *Anaerococcus lactolyticus*,* Actinobaculum schaalii*,* Anaerococcus obesiensis*,* Streptococcus anginosus*,* Varibaculum cambriense*, and *Propionimicrobium lymphophilum*, may play a role in the prostate cancer development [40, 42]. *Cutibacterium acnes* was prevalent in males diagnosed with prostate cancer [40, 43]. Gut microbiota link to prostate cancer or indirect pathway has also been investigated. Gut *Bacteroides massiliensis* had a higher prevalence among individuals diagnosed with prostate cancer, whereas *Faecalibacterium prausnitzii* and *Eubacterium rectale* were more prevalent in the healthy control males [41, 44].

Gut bacteria play a significant role in the metabolism and regulation of various hormones, including androgens. Androgens, such as testosterone and dihydrotestosterone (DHT), are crucial for the development and progression of prostate cancer. The gut microbiota can influence androgen levels through several mechanisms. Gut microbiota affects the conversion of dietary and endogenous compounds into bioactive molecules that affect androgen levels. Some bacteria produce enzymes that can convert steroids into androgens or their precursors [45]. Gut bacteria influence systemic inflammation and immune responses, which can, in turn, affect hormone levels. Chronic inflammation, driven by gut dysbiosis, has been linked to altered androgen levels and may impact prostate cancer progression [46]. Some gut bacteria are capable of synthesizing steroid hormones directly or influence the synthesis of androgens in the host [47].

Specific bacterial species were found to modulate the levels of key enzymes involved in steroid hormone synthesis, such as steroidogenic enzymes. This modulation can lead to changes in the levels of circulating androgens, which are critical in prostate cancer development [45]. Certain microbiota profiles might lead to decreased androgen levels, potentially affecting tumor growth and progression [46, 47].

Therefore, an imbalance in gut bacteria might lead to higher androgen levels, potentially increasing the risk of developing prostate cancer or accelerating its progression. Conversely, restoring a healthy gut microbiota might help regulate androgen levels and slow down cancer progression. Understanding the relationship between gut microbiota and androgen biosynthesis opens new avenues for prostate cancer treatment. Targeting gut microbiota to modulate androgen levels might provide a complementary approach to conventional therapies, such as hormone therapy.

## Mechanisms of microbiome–cancer interactions

The relation between the microbiome and cancer is controlled by many suggested mechanisms such as induction of host inflammatory pathways and oxidative stress, production of bacterial genotoxins, as well as microbial metabolites that facilitate the cancer development [11].

### Genotoxin production

Some bacteria secret toxins that affect the host DNA resulting in mutations in tumor suppressor genes or protooncogenes [48]. Toxins like colibactin, *Bacteroides fragilis* toxin (BFT), and trans 4-hydroxy-2-nonenal (4-HNE) are the most common. Colibactin is produced by polyketide synthase (*pks*)^+^ *E. coli*, and BFT is produced by enterotoxigenic *Bacteroides fragilis* [49]. Colibactin is mutagenic and causes breaks in the double strands, aneuploidy, and aberrant cellular division [50]. Cytolethal distending toxin (CDT) is another example of bacterial genotoxins. It is synthesized by enteric pathogenic strains like *Salmonella*,* Escherichia*, and *Campylobacter* spp. [51]. CDT consists of three subunits: CdtA, CdtB, and CdtC. They possess deoxyribonuclease activity and can induce DNA double-strand breaks, contributing to their carcinogenic properties. In the CRC mouse models, strains lacking CDT-secreting bacteria are less likely to develop cancer [52].

### Free radicals

The human gut is colonized by four primary phyla of commensal bacteria: Firmicutes, Bacteroidetes, Actinobacteria, and Proteobacteria. Firmicutes (*Lactobacillus*,* Streptococcus*,* Mycoplasma*, and *Clostridium*) and Bacteroidetes make up over 90% of the total bacterial load in the gut. The bacteria present in the gut influence the mitochondrial activity and thereby, change the cellular reactive oxygen species (ROS) levels and oxidative stress milieu. Communal bacteria secrete formylated peptides that specifically bind to G protein-coupled receptors (GPCRs) located on macrophages and neutrophils. This binding initiates an inflammatory response in the epithelial cells leading to the generation of superoxide radical by NADPH oxidase 1, enhancing the formation of ROS [53].

The administration of BFT enhances spermine oxidase activity which degrades spermine, resulting in an elevation in the ROS levels [54]. The gut epithelium is a rich source of nitric oxide (NO) due to the ability of gut *Lactobacilli* and *Bifidobacterium* to convert nitrate and nitrites into NO. Similarly, *Streptococcus* and bacilli form NO from L-arginine through the action of nitric oxide synthase (NOS) [53]. NOS produced by *Lactobacillus rhamnosus* induces the generation of ROS in the intestinal cells and promotes the proliferation of epithelial cells of mice [55].

In normal physiology, ROS serve as essential mediators in numerous cellular signaling pathways and immunological responses that occur spontaneously [54]. The nuclear factor kappa B (NF-κB) is a transcription factor involved in diverse functions such as regulating the activation of many genes implicated in cellular proliferation, viability, controlling cell cycle, and the development of drug resistance in many tumor types. NFκB also controls the expression of genes that control the level of ROS. In a feedback loop, ROS may either enhance or suppress the activity of NFκB signaling [56]. Furthermore, excessive ROS levels disrupt the balance of redox homeostasis leading to oxidative stress. Oxidative stress induces the process of oxidizing cellular constituents, such as DNA, lipids, and proteins, resulting in cell proliferation, angiogenesis, and metastasis [54, 56].

### Modification of signaling pathways in host cells

**Fig. 1:**
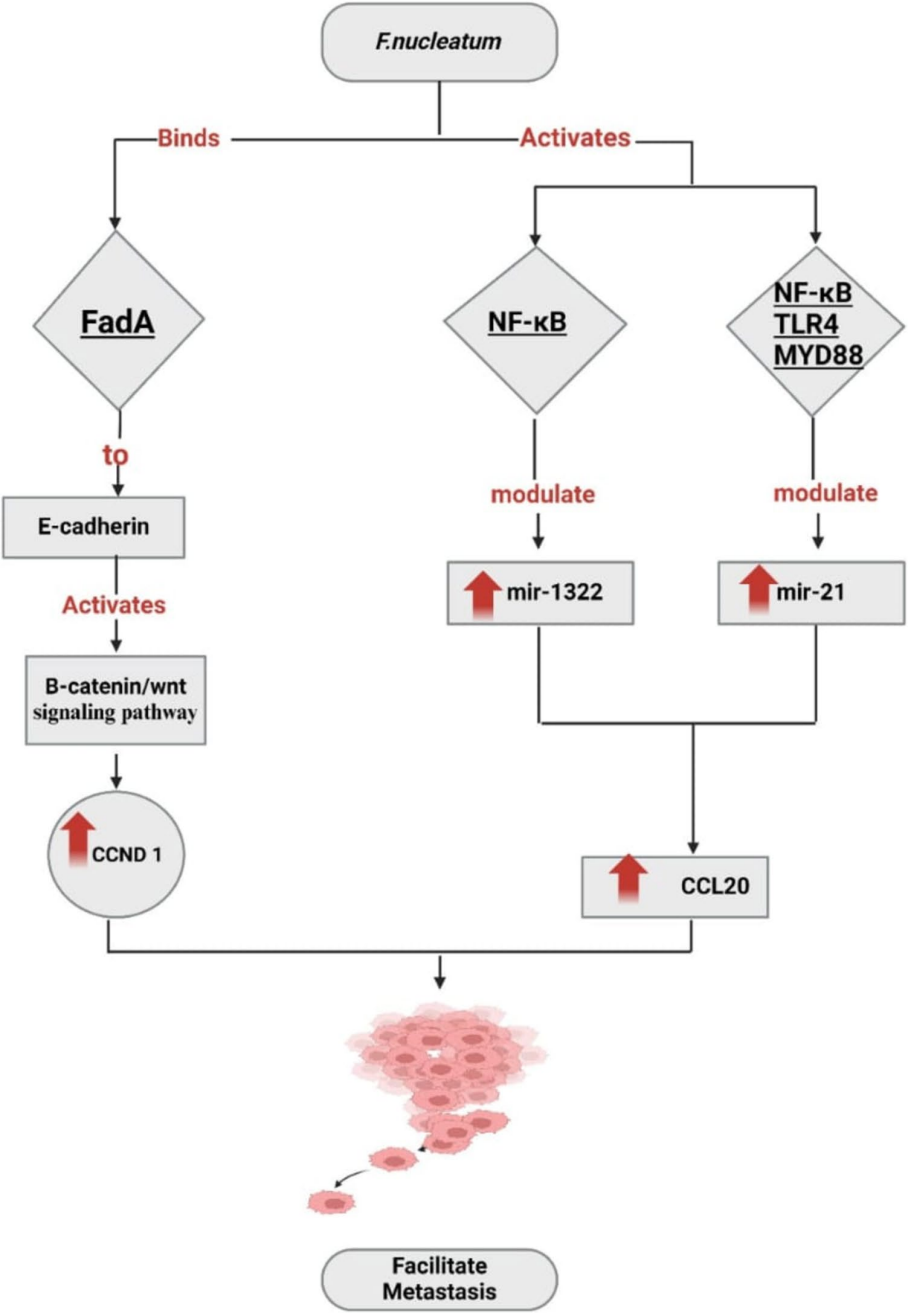
*Fusobacterium nucleatum* facilitates the metastasis of CRC via modulating the expression of FadA-Wnt/catenin signaling cascade and NF-κB/TLR4/MYD88 signaling pathway. *Created with BioRender.com*

Alterations in host cell signaling pathways play a significant role in the facilitation of several forms of cancer via the influence of gut bacteria, particularly *Fusobacterium nucleatum*. The initiation of the Wnt/β-catenin signaling cascade results from the binding of the FadA protein of *F. nucleatum* to E-cadherin in tissue (Fig. 1). This activation induces the nuclear translocation of catenin, culminating in the upregulation of inflammatory genes, Wnt, oncogenes (such as C-MYC), and cyclin D1 (CCND1) [12, 57]. The complex formed by FadA and E-cadherin includes the regulatory factor Annexin A1 (ANXA1), which is overexpressed in the CRC patients' sentinel lymph nodes (SLNs). ANXA1 is a ligand for the cell membrane's formyl peptide receptor 2 (FPR2) [58]. *F. nucleatum* did not induce Annexin A1 in non-cancerous SB cells but Annexin A1 induced only in the tumor mass's outer layer, indicating that the induction was limited to the malignant cells [60]. On CRC cells, the binding between *F. nucleatum* and E-cadherin initiates the β-catenin signaling pathway, leading to the upregulation of oncogenes such as cyclin D1 (CCND1) [59]. Cancer cells have upregulated ANXA1 expression on their membranes and demonstrate a strong affinity between FadA and E-cadherin compared to normal cells. The introduction of ANXA1 into non-cancerous cells resulted in an elevation in their aggressiveness and binding affinity. Emphasizing the significance, it is crucial to note that *F. nucleatum* has a promotional effect for the CRC via its interaction with E-cadherin, while concurrently impeding the proliferation of several other kinds of cancer cells [50].

Also, *F. nucleatum* influences CRC cells via the activation of the NF-κB signaling pathway. In the typical signaling pathway, active NF-κB consists of two subunits p50 and p65 (RelA) that form a heterodimer. Under normal conditions and without stimulation, this heterodimer is held inactive in the cytoplasm by IKB which is bound to p65 [60]. *Fusobacterium nucleatum* facilitates the dissemination of CRC via modulating the expression of miR-1322/CCL20 via the NF-κB signaling pathway (Fig. 1). The presence of *F. nucleatum* infection in the CRC cells resulted in elevated production of the chemokine CCL20. This upregulation of CCL20 was reported to facilitate the metastatic potential of CRC*.* This effect was validated in patient samples and animal models*.* Infection with *F. nucleatum* in CRC patients also activates TLR4/MYD88/NFκB signaling pathway, leading to upregulation of miR21 [61]. miR21 performs an essential role in promoting tumorigenesis [62]. Inhibition of miR21 reversed *F. nucleatum*'s oncogenic effects, suggesting that targeting miR21 could be a potential therapeutic target for the CRC. This pathway inhibits apoptosis leading to enhancing the survival of neoplastic cells, hence promoting cancer development.

In addition, the comprehensive activation of NF-κB in the intestinal cells upregulates the nitric oxide synthase which facilitates the breakdown of L-arginine, producing nitric oxide. Consequently, the deleterious effects of nitric oxide will affect the DNA integrity [54]. Silencing TLR4 or MYD88 inhibits NF-κB pathway and prevents the oncogenic effects of *F. nucleatum* in the CRC cells [62].

Another example is *H. pylori* bacteria which secrete a range of factors that can disrupt the normal functioning of the host's signaling pathways within the cell and make it easier for cancerous changes to occur. The key pathogenic elements among all factors related to virulence are CagA (cytotoxin-associated gene A) and its pathogenicity island (Cag PAI), and VacA (vacuolating cytotoxin A) [63]. The cagPAI contains genes that produce proteins responsible for constructing a type IV secretion system (T4SS). This system primarily interacts with α5ß1 and αvß6 integrins located on the plasma membrane of gastric epithelial cells, particularly through the CagL protein found at the end of the pilus. The process enables the direct delivery of many bacterial effectors such as CagA and pro-inflammatory components of the bacterial peptidoglycan, into the cytoplasm of the host cell [64].

**Fig. 2:**
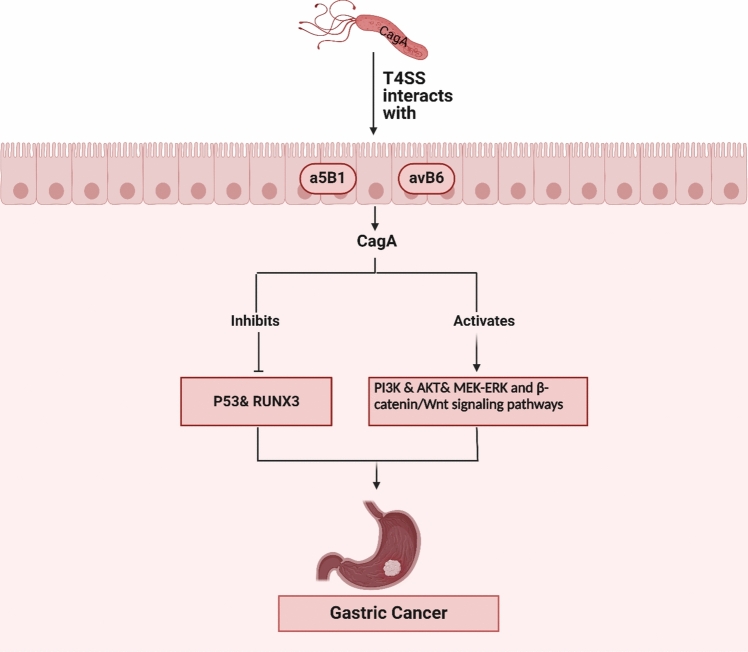
Effect of CagA on the epithelial cell’s growth and development of gastric cancer by activating certain signaling pathways. *Created with BioRender.com*

CagA exerts several different impacts on the epithelial cells (Fig. 2). This entails promoting cell growth by activating certain signaling pathways involved in cell division, such as the PI3 kinase–AKT28, 29, SHP2, GRB2, MEK–ERK, and β-catenin–WNT pathways. CagA also inhibits epithelial cell apoptosis by disrupting tumor suppressors such as p53 and RUNX3. CagA modifies the orientation of epithelial cells by directly interacting with the polarity protein MAP/microtubule affinity-regulating kinase 2 (MARK2 or PAR1b) and interferes with the formation and communication of cell junctions. The direct impact of CagA on epithelial cells may facilitate the progression of cancer, as evidenced by the development of carcinomas in transgenic mice and zebrafish that have been genetically modified to produce CagA, even in the absence of inflammation. Aside from its immediate impact on epithelial cells, CagA and the T4SS stimulate inflammatory NF-κB-dependent signaling which results in the attraction of inflammatory cells, induction of damage caused by ROS, and the activation of wound repair responses. All these processes have carcinogenic properties. The discovery of these results, along with the epidemiological evidence connecting CagA to the risk of gastric cancer, has resulted in the classification of CagA as a bacterial oncoprotein [64, 65].

### Modulation of drug metabolism

**Fig. 3:**
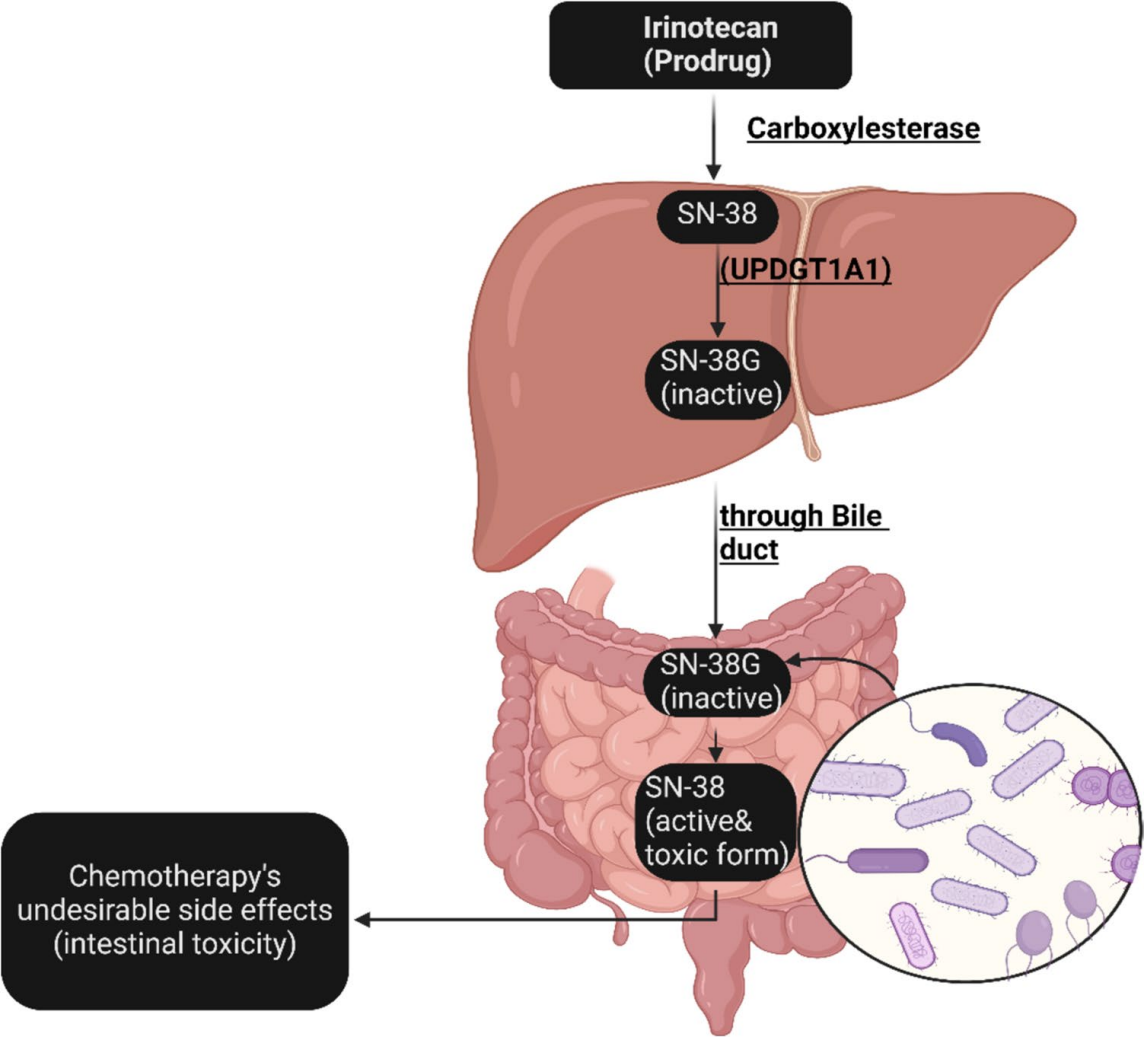
Undesirable side effects of the chemotherapeutic agent irinotecan by intestinal bacteria. *Created with BioRender.com*

Modulation of drug metabolism by microbiomes significantly influences drug efficacy and toxicity. For example, irinotecan is a pro-drug used as a chemotherapy for CRC patients. It is metabolized by carboxylesterases to a potent topoisomerase inhibitor SN-38. SN-38 is accountable for causing irreparable DNA harm [66]. This medication is a standard ingredient in cytotoxic combinations that are advised for CRC patients [67]. The liver transforms the active metabolite SN-38, through glucuronidation by UDPGT1A1 into an inactive form known as SN-38G, which is then discharged into the intestine through the bile duct [68]. Regrettably, the presence of bacteria in the colon results in the breakdown of SN-38G into its active and toxic form, SN-38, by β-glucuronidase (Fig. 3). Elevated blood levels of SN-38 lead to hematological toxicity (leukopenia and neutropenia) and damage to the intestinal epithelial cells, resulting in acute and life-threatening diarrhea [67].

Another example is Gemcitabine, a nucleoside analog, which is extensively employed as an anticancer medication in the therapy of multiple types of cancer such as bladder, ovarian, pancreatic, non-small-cell lung, and breast cancer [69]. Microbial enzymes facilitate the alteration of cytotoxic substances, rendering them non-functional. γ-Proteobacteria residing inside tumors influence the toxicity of gemcitabine [70]. They convert the drug into its inactive form, 2′,2′-difluorodeoxyuridine, which is catalyzed by the microbiome’s enzyme cytidine deaminase [71].

Cyclophosphamide (CTX) is among the clinically significant cancer drugs that, in part, derive their therapeutic efficacy from their capability to provoke immune responses against tumors [65]. Its function is greatly affected by the microbiome in the gut. In the tumor microenvironment, certain types of gram-positive bacteria including *Lactobacillus johnsonii*,* Lactobacillus murinus*, and *Enterococcus hirae* play a necessary role in inducing the CTX-derived immune responses [72]. The function of T helper 1 (Th1) and T helper 17 (Th17) cells is influenced by antibiotic treatment, which reduces the ability to treat P815 mastocytomas in mice receiving antibiotic treatment or in germ-free mice [68, 70].

**Fig. 4:**
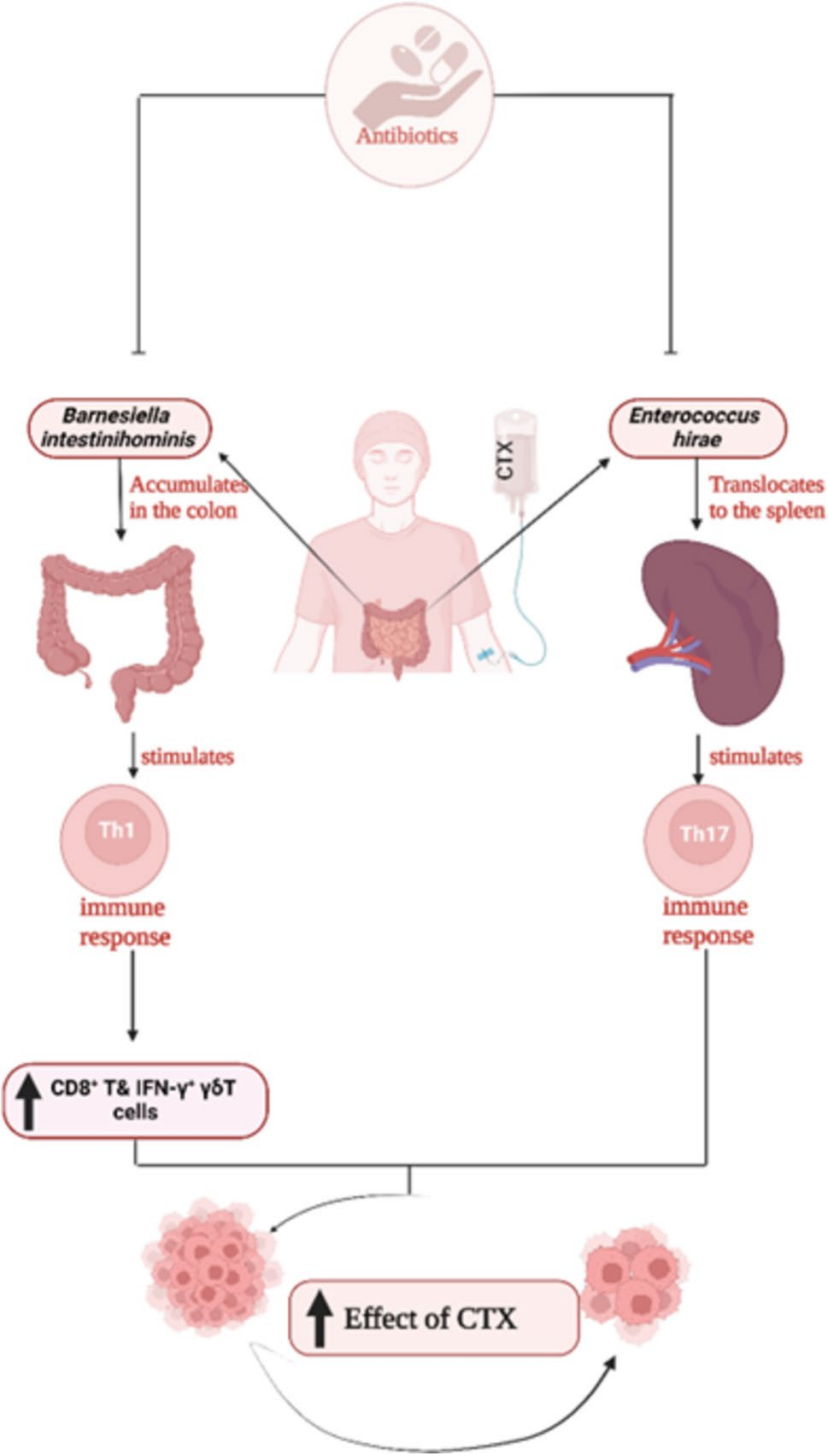
Effect of *Enterococcus hirae* and *Barnesiella intestinihominis* on inducing cyclophosphamide (CTX)-derived immune responses. *Created with BioRender.com*

Furthermore, *Enterococcus hirae* and *Lactobacillus johnsonii* efficiently translocate to the spleen to stimulate Th1 and Th17 immune responses, supporting the use of CTX. In mice inoculated with MCA205, injection of the antibiotic vancomycin (which fights gram-positive bacteria) also resulted in a decrease in CTX efficacy. Remarkably, CTX resistance is brought on by the depletion of gram-positive *Enterococcus hirae* and gram-negative *Barnesiella intestinihominis* (Fig. 4). Following CTX treatment, *Barnesiella intestinihominis* stimulates Th1 cell responses, increases the number of cytotoxic CD8+T cells, and encourages the infiltration of T cells that produce IFN-γ in the tumor microenvironment [70].

### Immune modulation

The immune system is made up of both adaptive immune cells like T and B lymphocytes and innate immune cells including mast cells, dendritic cells (DCs), natural killer cells (NK), neutrophils, and macrophages. These cells play a role in both preventing and promoting tumor development, with some cells having pro-tumor functions and others having antitumor functions. The immune system's ability to detect antigens from altered cells and produce memory and triggering cells is called “immunosurveillance.” The memory and triggering then actively search for and hinder the production of new tumor cells [73]. Tumor cells go through a three-step process called immunoediting, which involves: elimination, equilibrium, and escape. During elimination, the immune response to the tumor eradicates the first tumor cells. Then, the tumor enters a stable phase known as equilibrium, when certain tumor cells manage to evade the immune response, escaping the tumor elimination. Finally, the resilient clones successfully evade the body's immune system by developing characters that encourage tumor growth and lessen their capacity to elicit an immunological response. Consequently, tumors develop and the clinical symptoms emerge [73].

**Fig. 5:**
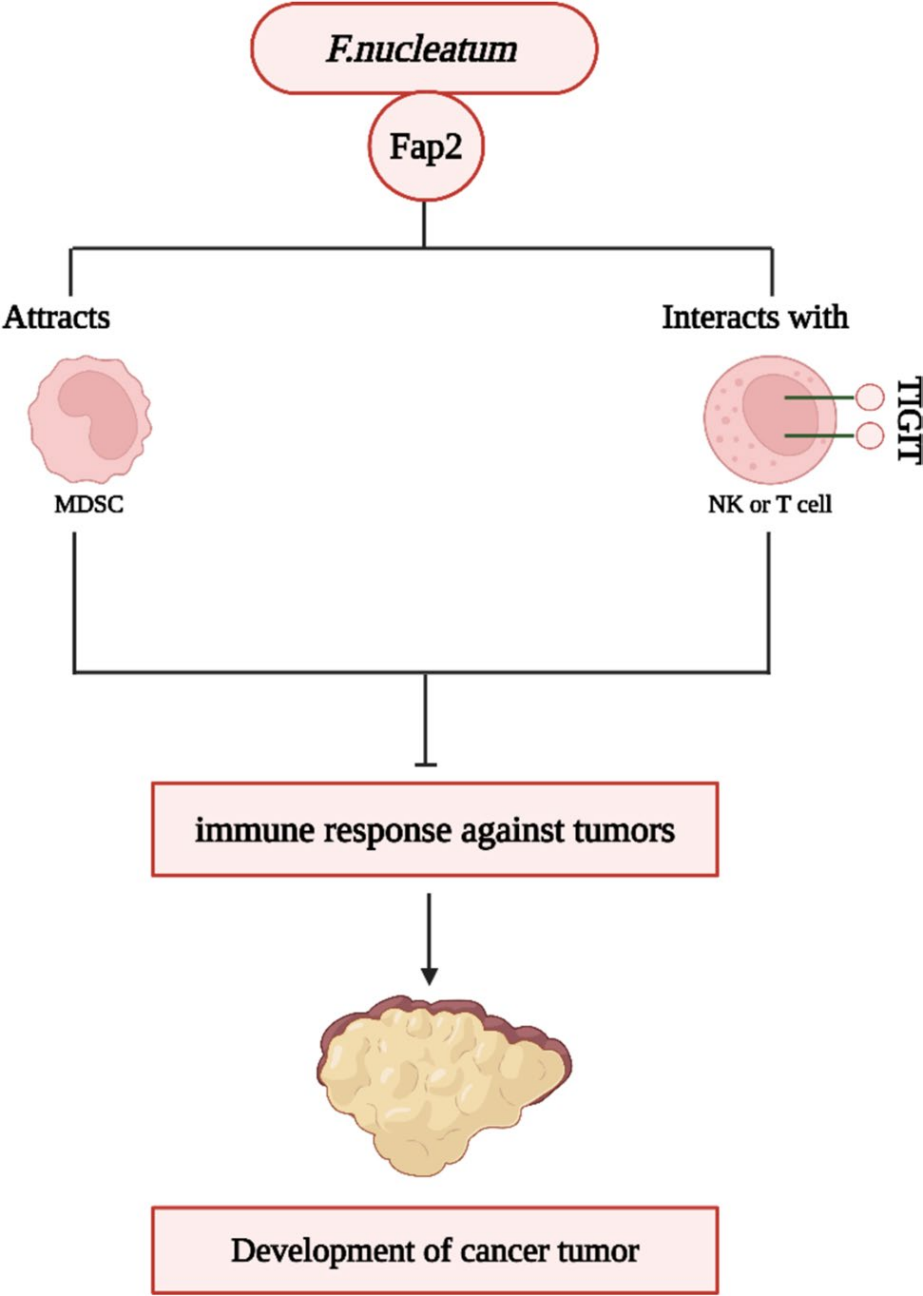
*Fusobacterium nucleatum* impedes the body's adaptive immune response mediated by anticancer T cells and hence is linked to the development of colorectal cancer (CRC). *Created with BioRender.com*

The mucosa-associated lymphoid tissue (MALT) and the intestinal commensal bacteria are the primary components of the intestinal immunological barrier. MALT is the primary constituent of the mucosal immune system, which encompasses gut-associated lymphoid tissue (GALT). It was shown that oral PSK, a polysaccharide generated from *Basidiomycetes* that is coupled to proteins, enhances the impaired immune response of CD4+T cells against tumors in the GALT of mice. This improvement is achieved by suppressing the synthesis of TGF-β and IFN-γ [74]. The regulation of immune system by microbes has been shown. An illustration of this phenomenon is found in the case of *F. nucleatum* and *Helicobacter pylori*, which can inhibit the functionality of T cells [41]. *Fusobacterium nucleatum* is further linked to the CRC and has been seen to impede the body's adaptive immune response mediated by anticancer T cells. The adhesion of *F. nucleatum* fibroblast activation protein 2 (FAP2) to the human T cell immunoglobulin and ITIM domain (TIGIT) produced on NK cells inhibits the NK cell functions allowing *F. nucleatum* to avoid the immune response against tumors (Fig. 5). *F. nucleatum* specifically attracts myeloid cells that infiltrate tumors, hence inducing inflammation in the tumor microenvironment, which supports the development of colon neoplasia. Myeloid-derived suppressor cells (MDSCs) were more abundant in ApcMin/ + mice that were given *F. nucleatum* compared to the control group. Furthermore, MDSCs were able to effectively suppress T cells. T lymphocytes identified specific bacteria, suggesting the potential for bacteria and tumor cells to have shared antigens [75].

Enterotoxic *Bacteroides fragilis* (ETBF) indirectly stimulates colonic epithelial cells (CECs) to produce chemokines and growth factors by interacting directly with IL-17 receptors. ETBF also stimulates the production of IL-17 in the submucosal layer. The combined action of IL-17 and altered CECs contributes to the growth of tumors via stimulating the STAT3 signaling pathway and preventing immunological effector cells. This process is facilitated by pro-angiogenic mediators, specifically matrix metalloproteinase-9 and vascular endothelial growth factor [74].

p53, a prominent tumor suppressor gene, is affected by *H. pylori*. When it malfunctions through mutation, p53 has been associated with the formation of many solid tumors including liver and gastric cancer. Prior research has demonstrated that *H. pylori* can control p53 via both mutational and non-mutational methods. Mutations leading to the inactivation of p53 have been documented in around 40% of gastric malignancies, with a higher prevalence observed in patients infected with the CagA positive strain of *H. pylori* [63]. Ralser et al., exposed wild-type C57BL/6 mice (WT) to an infection for a duration of 24 weeks and examined their immune responses. Compared to uninfected controls, there was a higher count of CD3+T lymphocytes within the epithelial layer of the small intestine and colon in *H. pylori*-infected mice. In contrast to the well-balanced immune profile observed in the stomach, there was a decrease in the number of Foxp3+Treg cells. Multiplexed ChipCytometry confirmed the general decrease in Treg cells and identified their localization within the lamina propria in infected colonic tissue. They provide evidence that *H. pylori* directly promotes the development of colon cancer by influencing the immune responses in the intestines and colon, as well as causing significant alterations in the microbiota and the balance of cells in the intestinal lining [76].

## Future directions and clinical application in the treatment of cancer by microbiome

### Fecal microbiota transplantation

Fecal microbiota transplant (FMT) involves the transplantation of fecal matter containing the bacteria and natural substances that fight against microorganisms, from a normal person to a sick person [77]. When compared to other tactics for modulation, FMT appears to offer multiple advantages over the alternatives. Although it enhances the variety of microorganisms and does not cause disturbance to the microbial balance in the gut like antibiotics do, its ability to establish itself in an ongoing manner also enables it to be formulated as a one-time treatment, providing therapeutic advantages over probiotics and prebiotics, which only seem to have temporary colonization [78]. It offers a promising approach to treating cancer by restoring the balance of microorganisms in the intestines, improving the way the body processes bile acids, and enhancing the effectiveness of immunotherapy [79]. The administration of FMT has the potential to increase the proportions of the beneficial bacterial genera *Lactobacillus*,* Butyricicoccus*,* Lachnoclostridium*,* Olsenella*, and *Odoribacter*, while reducing the proportions of the harmful genera *Helicobacter*,* Bacteroides*, and *Clostridium*. The complex changes in gut microbiota facilitate the growth of beneficial bacteria and inhibit the growth of pathogenic bacteria. This corrects the imbalances in the gut and regains a healthy intestinal environment [80].

FMT could potentially restore the efficacy of immune checkpoint inhibitors (ICIs) in patients previously resistant to these therapies, showing notable improvements in treatment responses [81]. Similarly, FMT enhanced the response to ICIs by modulating the gut microbiome, which is critical for improving treatment outcomes [82]. Changes in the gut microbiota appeared to influence systemic immune responses, which could enhance the efficacy of immunotherapy. Further research provides deeper insights into this phenomenon. For instance, FMT was reported to alter the gut microbiota in melanoma mice, leading to increased CD8+T cell activity and improved responses to anti-PD-1 immunotherapy. This shift in microbiota was associated with enhanced antitumor responses [83]. Similarly, FMT from immunotherapy responders significantly improved anti-PD-1 therapy effectiveness in resistant mice [83]. Successful responders showed a significant shift in their gut microbiota toward profiles more similar to those of the healthy donor microbiota. Additionally, FMT effectively managed ICI-induced colitis by restoring a balanced gut microbiota and alleviating colitis symptoms [82]. Some patients experienced partial or complete tumor regression following FMT. Collectively, these studies underscore the promising potential of FMT as a strategy to overcome resistance to immunotherapy and address its associated complications. However, further research is needed to validate these results, understand the underlying mechanisms, and optimize the safe use of FMT in clinical settings.

### Probiotics, prebiotics, and cancer

Supplementing the diet with live microorganisms, or probiotics, is a good strategy to change the composition of the gut microbiota. Probiotics are defined as live microorganisms, typically bacteria or yeasts, that confer a health benefit to the host when administered in adequate amounts. When ingested, probiotics contribute positively to the balance of the gut microbiota and enhance overall health. They function through various mechanisms, such as improving gut barrier function, modulating the immune system, and outcompeting pathogenic microbes. In addition, they eradicate pathogenic bacteria, preserve the intestinal barrier integrity, regulate the bile acid metabolism, control intestinal motility and gas production, synthesize vitamins, produce short-chain fatty acids (SCFAs), enhance the host's immune system, and exert anticancer effects [84], thereby supporting digestive health and potentially benefiting other aspects of the host’s well-being. *Lactobacilli* and *Bifidobacteria* are the most widely used probiotics [84].

Lactobacillus produces reduced glutathione (GSH) as well as many antioxidant enzymes such as superoxide dismutase (SOD) and catalase. It also produces antiangiogenic factors, reduces inflammation and DNA degradation, and decreases tumor volume. In addition, it inhibits the production of some proteins and polyamine constituents linked to the development of cancer. These actions help in the prevention and treatment of cancer [85]*. Bifidobacterium* arrests the cell cycle at G0/G1 phase in the CRC and increases the expression of alkaline phosphatase, which is downregulated in the cancerous tumor cells. *Bifidobacterium* induced the innate immune system to prevent the development of glioblastoma tumors. This bacterium promotes the development of dendritic cells and tumor-specific CD8+T lymphocytes, enhancing their ability to combat tumor growth. Additionally, the initiation of type I Interferon (IFN-I) signaling also impacts the regression of tumors [84]. *Bacillus polyfermenticus* was first used in 1993 to treat intestinal diseases. It produces bacteriocin and inhibits the growth of certain cancer cell types, such as lung, cervical, breast, and colon cancer. Moreover, it prevents the colonization in HT-29 and Caco-2 cell lines [85].

Inulin and galacto-oligosaccharide, commonly used prebiotics, can increase the presence of *Bifidobacterium*,* Lactobacillus*, and *Faecalibacterium spp*. in the intestines of humans. These bacteria are known to enhance the body's ability to fight against cancer by improving the immune response [86]. The word "prebiotic" is a type of nutrition that is indigestible and exerts a positive impact on its host by specifically promoting the proliferation and/or action of a small number of bacteria in the colon, hence enhancing the health of the host [87]. The primary role of prebiotics is to effectively enhance the activity of the existing metabolic processes in the colon. Prebiotics are short-chain carbohydrates that do not break down in the upper portion of the digestive tract. Prebiotics are the main substances in the colon that bacteria use to proliferate [83]. There are different types of prebiotics, one of them is galacto-oligosaccharides (GOS) which is a category of carbohydrates consisting of oligo-galactose, together with lactose and glucose. GOS exist naturally in milk and are also produced through the transgalactosidase activity of β-galactosidases. GOS molecules are not broken down in the intestine because the necessary enzymes β-galactosidase and beta-glucosidase are absent. The colonic bacteria metabolize the GOS to generate SCFAs, lactate, acetate, and gases, promoting more frequent bowel movements. The presence of GOS in breast milk provides protection against gastrointestinal pathogenic germs for infants who are breastfeeding. GOS also increase the amounts of IL-8, IL-10, and C-reactive protein in the serum, but decrease the IL-1β level. GOS administration also boosts NK cell activity [84]. Additionally, GOS lower the amount of lithocholic acid, a secondary bile acid, in the feces. This leads to an increase in the fecal pH and a decrease in the activity of nitroreductase and β-glucuronidase. Prebiotics such as fructooligosaccharides, lactulose, and β-(1-4) GOS are important in preventing the CRC [88].

### Microbiome-based biomarkers for early detection and prognosis

The initiation of the Human Microbiome Project (HMP) in 2007 aimed to identify the connections between alterations in the microbiota during states of well-being and illness, while also creating a uniform reference point. This project found that the study, monitoring, or alteration of the human microbiome can improve human health. The study of the microbiome has mostly depended on the use of 16S ribosomal RNA, which has revealed a significant diversity in the makeup of the typical microbiota among various people. However, there are caveats associated with this technique, and efforts lead to the building of genetic databases derived from extracted DNA specimens, or metagenomics [89]. The modified composition of circulating microbiota and decrease in diversity was first linked to hepatocellular carcinoma (HCC) in 2019 [90]. They also confirmed the diagnostic precision of the blood microbiome in diagnosing HCC. Ren et al. observed a decline in the butyrate-producing bacteria genera *Ruminococcus*, *Oscillibacter*, *Faecalibacterium*, *Clostridium* IV, and *Coprococcus* in their study on patients with early HCC. On the other hand, in comparison with the control group, they reported a rise in the levels of *Klebsiella* and *Haemophilus*, which are known to produce lipopolysaccharide. These findings suggest that the gut microbiota–liver relationship may be used to monitor and prevent the development of HCC by targeting the altered gut microbiota [91]. The concept of a “blood microbiota” refers to the potential presence of microbial communities or microbial DNA in the bloodstream. Recent studies have detected microbial DNA in blood samples, suggesting that microorganisms might transiently or persistently exist in the circulatory system. However, the interpretation of these findings remains controversial, with some researchers arguing that such detections reflect contamination rather than a true microbiota [92]. It is crucial to establish robust methodologies to ensure that findings are reproducible and reflect meaningful biological phenomena rather than artifacts.

Zackular et al., developed a classification model using 90 fecal samples: 30 from CRC patients, 30 from colonic adenoma patients, and 30 from healthy individuals. They found substantial differences in the gut microbiota between healthy individuals and those with adenoma or CRC. In addition, fecal microbial screening showed a 0.798 AUC predictive accuracy in detecting CRC. They accomplished a significant improvement in the discriminatory power of fecal microbial screening among the three clinical groups, surpassing the performance of risk factors, by combining their gut microbiome data with established clinical risk variables, such as body mass index, age, and race [93, 94].

## Summary

The intensity and diversity of microbiome have substantial effects on the cancer development, progression, and response to therapy. The complex relationship between the microbiome and many types of cancer, such as colorectal, lung, prostate, and breast cancer, is a hot research area. Changes in the composition of the gut microbiota, and release of metabolites that induce inflammation and disrupt the immune system are factors that could initiate colon, lung, and prostate cancer. Research on breast cancer microbiota indicates that microorganisms may have an impact on inflammation and immunological responses in breast tissue, therefore affecting the milieu in which tumors develop. The microbiome–cancer connection is driven by many mechanisms such as the creation of genotoxins, oxidative stress, modification of signaling pathways, drug metabolism, and immunological modulation. These mechanisms offer potential targets for therapeutic interventions. Future advancements focus on precision medicine techniques, therapeutics based on the microbiome, integration with immunotherapy, and the discovery of biomarkers that will help in the early detection and diagnosis of cancer. These developments will pave the way for personalized strategies in cancer care that utilize the mutually beneficial relationship between microbial communities and the host's physiology.

## Challenges and future prospectives

While the potential of microbiome profiling in cancer research is promising, several significant challenges and controversies need to be addressed. One major challenge is distinguishing whether observed links between microbiome profiles and cancer are indicative of a causal relationship or merely correlational. Determining causality requires rigorous experimental designs, including longitudinal studies and mechanistic research, to establish whether changes in the microbiome directly contribute to cancer development or are a consequence of the disease. There is currently no consensus on what constitutes an optimal microbiome diagnostic. The variability in microbiome composition across individuals, coupled with differences in sequencing technologies and analytical methods, complicates the development of standardized diagnostic criteria. Robust, reproducible biomarkers are needed to ensure that microbiome diagnostics can reliably predict cancer risk and progression. The effectiveness of microbiome diagnostics hinges on the quality of sequencing technologies and the clarity of signals they provide. While advances in high-throughput sequencing have improved our ability to analyze microbial communities, challenges remain in achieving sufficient resolution and accuracy to differentiate between relevant and incidental microbial changes. Recent controversies in microbiome diagnostics highlight the need for cautious interpretation of results. For instance, a paper published in *Nature* that reported on blood and tissue microbiome diagnostics was subsequently retracted due to issues with data integrity and reproducibility [https://www.nature.com/articles/s41586-020-2095-1]. This underscores the importance of critical evaluation and validation of findings in microbiome research before they are translated into clinical practice.

## Data Availability

No datasets were generated or analyzed during the current study.
